# Pentalogy of Fallot with Anorectal Malformation: A Case Report

**DOI:** 10.31729/jnma.8705

**Published:** 2024-08-31

**Authors:** Sunil Raja Manandhar, Pankaj Kafle, Asish Lal Shrestha, Dipendra Rai

**Affiliations:** 1 Neonatology Division, Department of Pediatrics, Kathmandu Medical College and Teaching Hospital, Sinamangal, Kathmandu, Nepal; 2 Department of Radiology, Kathmandu Medical College Teaching Hospital, Sinamangal, Kathmandu, Nepal; 3 Department of Pediatric Surgery, Kathmandu Medical College and Teaching Hospital, Sinamangal, Kathmandu, Nepal

**Keywords:** *anorectal malformation*, *case report*, *congenital cyanotic heart disease*, *pentalogy of Fallot*

## Abstract

Pentalogy of Fallot is a rare form of congenital cyanotic heart disease with a prevalence of 3/10,000 live births characterized by an association of Tetralogy of Fallot with Atrial Septal Defect. Pentalogy of Fallot with anorectal malformation is also a rare combination. Here we describe one of the rare case reports of a full-term, 38 weeks, female baby diagnosed with pentalogy of Fallot with imperforate anus and rectovaginal fistula at a tertiary care hospital. Pentalogy of Fallot combined with an imperforate anus and rectovaginal fistula is an exceptionally rare and complex congenital condition. The co-existence of these anomalies emphasizes the need for thorough prenatal and postnatal evaluation for early detection and management.

## INTRODUCTION

Pentalogy of Fallot (POF) is a rare form of congenital cyanotic heart disease with a prevalence of 3/10,000 live births characterized by an association of Tetralogy of Fallot (TOF) with atrial septal defect (ASD).^1^ It consists of ventricular septal defect (VSD), overriding of aorta, right ventricular hypertrophy (RVH), pulmonary stenosis, and ASD with right to left intra-cardiac shunting of blood causing decreased pulmonary blood flow and cyanotic episodes in-between.^2^ We describe a rare case of female term baby presented with anorectal malformation [imperforate anus (IA) and rectovaginal fistula] and diagnosed as POF at 2 hours of life at a teaching hospital by bedside echocardiography and reconfirmed by Computed Tomography (CT) Angiography after 24 hours of life.

## CASE REPORT

A 38 weeks, full-term, female baby with a birth weight of 3.0 kg was delivered at a tertiary care hospital via elective cesarean section with APGAR scores 7/10 and 9/10 at 1 min and 5 min respectively to 29 years old G_2_ P_1_ L_0_ A_1_ mother. The baby was born to non-consanguineous parents with a maternal history of gestational diabetes mellitus (GDM) and hypothyroidism. She had been taking thyroxine since the preconception period. Immediately after birth during head-to-toe examination, an imperforate anus was noticed. Antenatal fetal echocardiogram was not done.

**Figure 1:**
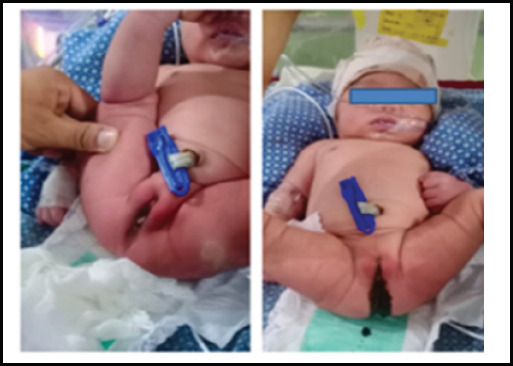
Showing imperforate anus and passing meconium per vagina suggestive of rectovaginal fistula.

The baby was admitted to Neonatal Intensive Care Unit (NICU) for assessment of other associated congenital malformations. Physical examination showed central cyanosis; preductal oxygen saturation in room air was 82% without any signs of respiratory distress. Passage of meconium was seen from the vaginal opening suggestive of IA (low type) with rectovaginal fistula (Figure 1).

On cardiac examination, a loud pansystolic murmur grade IV was heard over the left parasternal area at the fourth intercostal space. Other systemic examinations were within normal limits.

**Figure 2:**
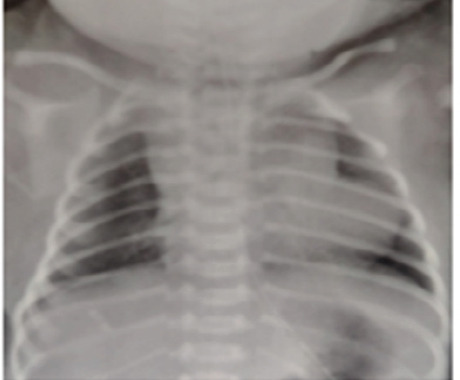
Chest X-ray showing boot shaped heart.

**Figure 3:**
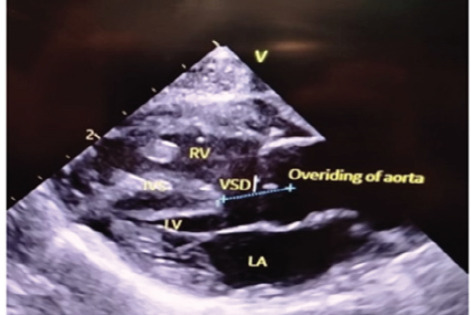
Echo PLAX view showing Large VSD, hypertrophied right ventricle with overriding of aorta.

The chest X-ray showed a boot-shaped heart (Figure 2) and echocardiography done at the second hour of life showed a large malaligned 9 mm VSD with left-to-right shunt and overriding of aorta along with hypertrophied right ventricle noted in the parasternal long axis (PLAX) view (Figure 3).

**Figure 4:**
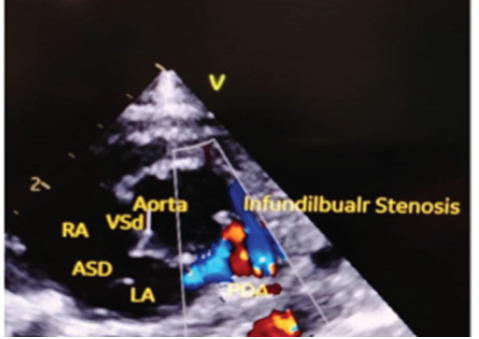
Echo PSAX view showing ASD, VSD, and severe infundibular stenosis of Pulmonary artery.

**Figure 5:**
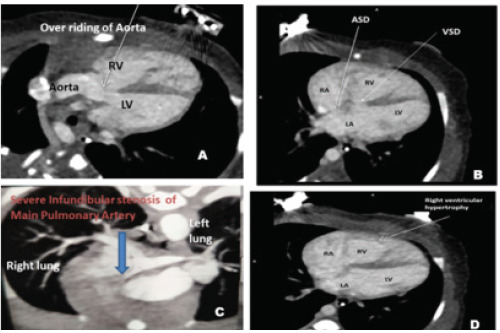
CT Angiogram showing overriding of aorta (A), Atrial septal defect and Ventricular Septal Defect (B), Severe infundibular stenosis of main pulmonary artery (C) and Right ventricular hypertrophy.

Parasternal short axis (PSAX) view showed severe infundibular stenosis of pulmonary artery, ASD and patent ductus arteriosus (PDA) with color doppler showing left-to-right shunt both (Figure 4). So, the findings were suggestive of POF with anorectal malformation (imperforate anus with rectovaginal fistula). A skeletal survey did not detect any vertebral anomalies while renal anomalies were ruled out through renal ultrasound. A cardiac CT angiography done on the second day of life also showed similar findings as that of echocardiography in the form of severe pulmonary infundibular stenosis of pulmonary artery, RVH, overriding of aorta, large VSD and ASD (Figure 5), findings suggestive of POF.

During NICU stay, the neonate received Inj. Milrinone to reduce pulmonary artery systolic pressure and to maintain cardiac support. She was kept on Inj. Prostaglandin E1 in the form of continuous intravenous (IV) infusion to maintain the ductal patency of the PDA medically. On the fourth day of life, a colostomy procedure was performed for initial management of anorectal malformation. However, due to a deteriorating clinical condition characterized by persistent severe hypoxemia, the neonate required mechanical ventilation on the sixth day of life. Although a cardiac surgical option was considered, however due to multiple cardiac and anorectal malformations, receiving very guarded prognosis, parents opted not to have cardiac surgery for the baby and preferred for a conservative palliative management only. Unfortunately, on the eleventh day of life, the baby developed progressive hemodynamic instability, poor peripheral perfusion with persistent desaturation with SPO_2_ of 60%. Echocardiography was repeated which revealed a shunt reversal (right-to-left) at the level of large VSD, which was left-to-right during the initial few days of life. Unfortunately, the baby expired on the twelfth day of life.

## DISCUSSION

POF is characterized by antero-cephalad deviation of the infundibular septum, resulting in a malalignment type of nonrestrictive perimembranous VSD, overriding aorta, RVH and right ventricular outflow obstruction in the form of infundibular stenosis of pulmonary artery. An additional ASD is seen in 3-5% of cases, with ostium secundum type being the most common.^3^ In our case also there was malaligned VSD, overriding aorta, RVH with severe infundibular stenosis of pulmonary artery along with anorectal malformation. POF is one of the rare congenital heart diseases and POF associated with anorectal malformation in the form of IA with rectovaginal fistula is even very uncommon.

One of the rare cases reported by Suhail et al. in India described a full term female baby with birth weight 2.5 kg with POF having five cardiac anomalies.^4^ The echocardiogram examination revealed overriding aorta, RVH, pulmonary stenosis, ASD (right-to-left shunt) and VSD (bidirectional flow). There was a history of rubella infection in mother. This baby had PDA also and developed peripheral cyanosis and required NICU care. Due to indecisive parents reluctant for neonatal cardiac surgery, the baby expired before cardiac surgery. Apart from POF, other congenital malformations were not noted. However, in our case, the female baby had IA with rectovaginal fistula apart from POF. The parents of the baby in our case were also reluctant undergoing cardiac surgery due to its guarded prognosis and it is still difficult to save these types of babies, particularly in low-middle-income countries like Nepal where neonatal cardiac surgical infrastructure is suboptimal.

In various case studies, there was association between cardiac anomalies with gastrointestinal malformations. Most common cardiac defects mentioned were ASD, PDA and Pulmonary stenosis according to study done by Kamal et al.^5^ Voisin et al. reported that babies with congenital heart disease have a much higher incidence of intestinal malformations as compared to babies with normal heart. Among these babies, the incidence of congenital heart diseases is 9-14% with VSD and TOF.^6^

This case highlights the challenges and critical considerations in managing complex congenital conditions, emphasizing the importance of early detection, a coordinated care approach, and ongoing research and documentation.
